# Automatic polyp detection and measurement with computed tomographic colonography: A phantom study

**DOI:** 10.2349/biij.5.3.e15

**Published:** 2009-07-01

**Authors:** S Virmani, AS Lev-Toaff, LM Ciancibello

**Affiliations:** 1 Philips Healthcare, Ohio, United States; 2 Department of Radiology, Hospital of the University of Pennsylvania, Philadelphia, United States

**Keywords:** Polyp measurement, CT colonography, CAD, automatic measurement

## Abstract

**Purpose:**

The purpose of this study is to assess the performance of computer-aided detection (CAD) software in detecting and measuring polyps for CT Colonography, based on an in vitro phantom study.

**Material and methods:**

A colon phantom was constructed with a PVC pipe of 3.8 cm diameter. Nine simulated polyps of various sizes (3.2mm-25.4mm) were affixed inside the phantom that was placed in a water bath. The phantom was scanned on a 64-slice CT scanner with tube voltage of 120 kV and current of 205 mAs. Two separate scans were performed, with different slice thickness and reconstruction interval. The first scan (thin) had a slice thickness of 1mm and reconstruction interval 0.5mm. The second scan (thick) had a slice thickness of 2mm and reconstruction interval of 1mm. Images from both scans were processed using CT Colonography software that automatically segments the colon phantom and applies CAD that automatically highlights and provides the size (maximum and minimum diameters, volume) of each polyp. Two readers independently measured each polyp (two orthogonal diameters) using both 2D and 3D views. Readers’ manual measurements (diameters) and automatic measurements from CAD (diameters and volume) were compared to actual polyp sizes as measured by mechanical calipers.

**Results:**

All polyps except the smallest (3.2mm) were detected by CAD. CAD achieved 100% sensitivity in detecting polyps ≥6mm. Mean errors in CAD automated volume measurements for thin and thick slice scans were 8.7% and 6.8%, respectively. Almost all CAD and manual readers’ 3D measurements overestimated the size of polyps to variable extent. Both over- and underestimation of polyp sizes were observed in the readers’ manual 2D measurements. Overall, Reader 1 (expert) had smaller mean error than Reader 2 (non-expert).

**Conclusion:**

CAD provided accurate size measurements for all polyps, and results were comparable to the two readers' manual measurements

## INTRODUCTION

Colorectal cancer (CRC) is the second leading cause of cancer death in the United States. The American Cancer Society estimates that about 148,810 new cases of colorectal cancer will be diagnosed in 2008. In the vast majority of cases, colorectal cancer develops slowly from precancerous polyps and can be prevented if precancerous polyps are removed. This indicates the importance of screening for colorectal cancer. However, patient compliance with screening recommendations remains low, due, at least in part, to the limitations of current screening techniques (e.g. optical colonoscopy, flexible sigmoidoscopy, barium enema and fecal occult blood test etc.).

More than a decade ago, CT Colonography was introduced as a non-invasive technique for the detection of colonic polyps and colorectal cancer. Since then tremendous advancements have occurred, including improvements in the examination technique itself and also in the interpretation methods. CT Colonography studies can be read primarily using 3-dimensional visualisation techniques with 2-dimensional images used for lesion characterisation or by means of primary 2-dimensional reading for detection and characterisation. Polyp size is the most important criteria for assessing the risk of malignancy and the need for follow up in CT Colonography (CTC). Size was used as the most important criterion for risk stratification of polyps during the development of CT Colonography: Reporting & Data System (CRADS) [1]. Diameter (maximum linear dimension) has been the standard parameter for reporting polyp size. Based on size (maximum diameter), polyps are typically classified into three categories: Diminutive (≤ 5 mm), Intermediate (6-9 mm) and Large (≥ 10 mm). Recommendations for patient follow-up change significantly, based on the number and more importantly, the size category of polyps that are detected. Therefore, accurate and reproducible diameter measurement both during 2D and 3D interpretation of CTC studies is critical. Polyp volume has also been proposed as a better measure than linear dimension or diameter [2]. Further investigational studies are needed to study the value of polyp volume compared to maximum diameter. During the past decade, several studies [3-5] have demonstrated high sensitivity and specificity of CTC for polyp detection but few other studies [6, 7] have questioned that and created disparity in the results. For widespread acceptance of CTC, methods to improve its accuracy and reproducibility are required. Computer Aided Detection (CAD) has been proposed as a possible solution [8]. CAD techniques not only provide the capability of detecting polyps but also provide automatic measurement of the volume and diameter of polyps.

## MATERIALS AND METHODS

The primary objective of our study was to evaluate the accuracy of automatic diameter and volume measurements provided by Computer Assisted Detection (CAD) software (Extended Brilliance Workspace, Philips Healthcare, Andover, Massachusetts) for polyp-simulated structures in a colon phantom. The secondary objective includes comparison of 2D and 3D manual measurements performed by two human readers with the automatic measurements of CAD. Furthermore, we sought to assess the effect of scanning parameters (slice thickness and reconstruction interval) on both automatic and manual measurements.

### Colon Phantom

**Figure 1:**
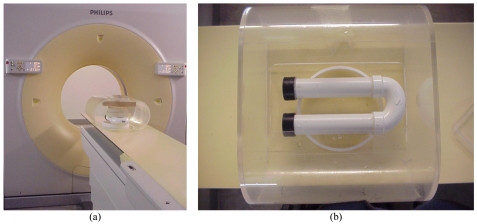
(a) Experimental Set-up: Colon phantom submerged in water placed on the scanner table (Brilliance 64, Philips Healthcare, Andover, Massachusetts, USA); (b) Close-up view of the colon phantom inside the hollow container.

**Figure 2:**
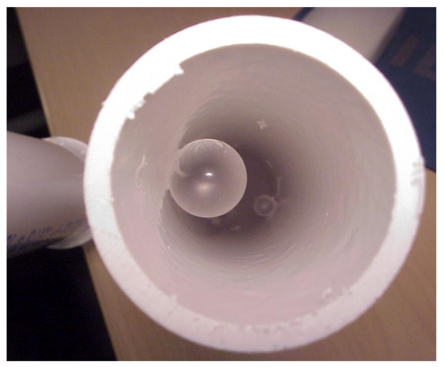
Glass beads (to mimic polyps) glued to the inner surface of the colon phantom.

A colon phantom was constructed using a 1.5" diameter PVC pipe. Ten phantom polyps comprised of glass beads of various diameters were glued to the inner surface of the pipe to mimic polyps on the colon wall (Fig 2). A very thin layer of glue was applied to the phantom polyps to avoid any over-estimation of the polyp size by the software. The glass beads were chosen as the phantom polyps because their CT density was in a similar range to real polyps, which vary between 20-100 HU [9]. The phantom was submerged in water as shown in Fig 1 to simulate the attenuation of the x-ray beam by the soft tissues surrounding the colon in vivo. The phantom was sealed at both ends to avoid any water flowing into the tube. Wooden planks were used to hold the submerged hollow phantom in place in the water bath.

### Phantom Polyps

**Figure 3:**
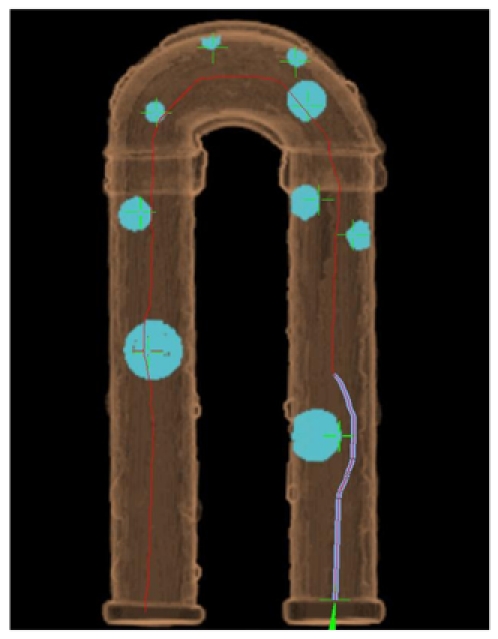
Placement of all polyps shown in a 3-dimensional overview image reconstructed from CT data.

The phantom polyps were spherical in shape and were of different sizes. The reference diameter of the phantom polyps were measured using mechanical calipers and their volume was mathematically calculated. The distribution of the phantom polyps by diameter and their classification into clinically relevant polyp size classes are provided in Tables 1 and 2 respectively. The placement of the polyps within the phantom is shown in Figure 3. The polyp sizes were chosen such that there is at least one representative phantom polyp from each of the standard polyp size categories: diminutive (≤ 5 mm), intermediate (6-9 mm) and large (≥ 10 mm).

**Table 1:** Phantom polyps used in the colon phantom with their actual diameters measured using mechanical calipers

Actual Diameter (mm)	Quantity
25.4	1
22.2	1
15.9	1
12.7	2
9.6	1
6.3	3
3.2	1

**Table 2:** Defining the clinically relevant ranges of polyp size. Recommended patient follow-up changes substantially for polyps detected in the higher size ranges.

Diameter Range	Category	Number of phantom polyps
< 6 mm	Small	1
6-9 mm	Intermediate	4
≥ 10 mm	Large	5

### Acquisition Protocol

The phantom was scanned on a Brilliance 64-slice CT scanner (Philips Healthcare, Andover, Massachusetts, USA). The scan was performed in a helical mode at a collimation of 64x0.625, 120 kV, pitch of 0.95 and 205 mAs. The data was reconstructed into thick and thin images using slice thickness of 2 mm/ reconstruction interval of 1 mm and slice thickness 1 mm/reconstruction interval 0.5 mm, respectively. This was done to analyse the effect of slice thickness on the accuracy of manual and automatic measurements.

### Human readers for manual measurements

For manual measurements, two readers, both familiar with the CT colonography software but with different levels of experience, were employed. Reader 1 was an abdominal radiologist with 22 years of experience in radiology and fellowship training in gastrointestinal imaging, and was also an expert in CT Colonoscopy (4 years of experience in CT Colonoscopy and read over 200 cases at the time of the study). Reader 1 was also an expert with the workstation and software used in the study. Reader 2 was a radiologic technologist with no clinical experience interpreting CT Colonoscopy but with more than 5 years of experience in advanced CT post-processing and 3D imaging. The two readers interpreted the images separately and were blinded to the results from each other and also to the ground truth.

### Image Processing and Interpretation

The reconstructed images of the phantom were transferred to a clinical post-processing workstation (Extended Brilliance Workspace, Philips Healthcare, Andover, Massachusetts, USA). The workstation includes an advanced Virtual Colonoscopy application with varied displays including 2D multi-planar, endoluminal and a Perspective-Filet View (dissection view) to optimize interpretation and Colon CAD software for automatic detection, segmentation and measurement of polyps.

The Colon CAD software (Philips Healthcare, Andover, Massachusetts, USA) used in the study is a feature-based technique that identifies potential polyps based on morphology and density. The CAD algorithm performs this operation in three steps. First, the algorithm identifies convex elevated regions with positive curvature throughout the colonic surface. Second, it calculates the likelihood value based on morphology (including size, convexity and compactness) and Hounsfield Unit (intensity) average and standard deviation of each of those candidates. Finally, a subset of those candidates are classified and highlighted as possible polyps based on a pre-defined threshold for the likelihood value and on an optimal setting on the Free-receiver operating curve (FROC).

**Figure 4:**
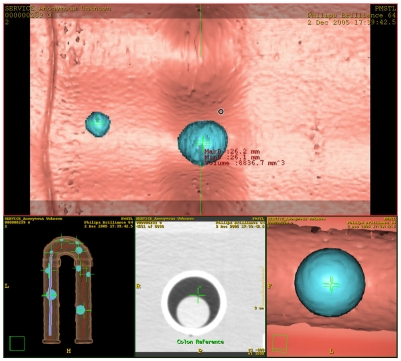
Colon Computer Aided Detection (CAD) software automatically detecting and measuring the polyp diameters and volume.

Both phantom scans were processed by the Colon CAD algorithm. Automatic diameter (two orthogonal) and volume measurements provided by CAD were recorded. An example of CAD detection and automatic measurement is shown in Figure 4. Both readers read the scans using a combination of perspective filet view and endoluminal 3D view, making measurements on 2D images and 3D endoluminal images on separate occasions. 3D measurements were made on the endoluminal view and 2D measurements were made on either the axial, coronal or sagittal 2-dimensional reconstructed images (whichever provides the optimized “maximal” dimension), as in clinical practice. A substantial time gap (approximately 1 month) was built in between 2D and 3D measurements to avoid recall bias.

### Statistical Analysis

The accuracy of measurements from CAD and readers (2D and 3D) was measured using absolute mean error (%) calculations compared to the actual measurements of the simulated-polyps. A Student T Test was used to compare the volume and diameter measurements from CAD and readers, between thick and thin slices. Bland-Altman Analysis [10] was used to evaluate the inter-observer agreement between the readers for the repeated manual measurements (2D and 3D).

## RESULTS

**Figure 5:**
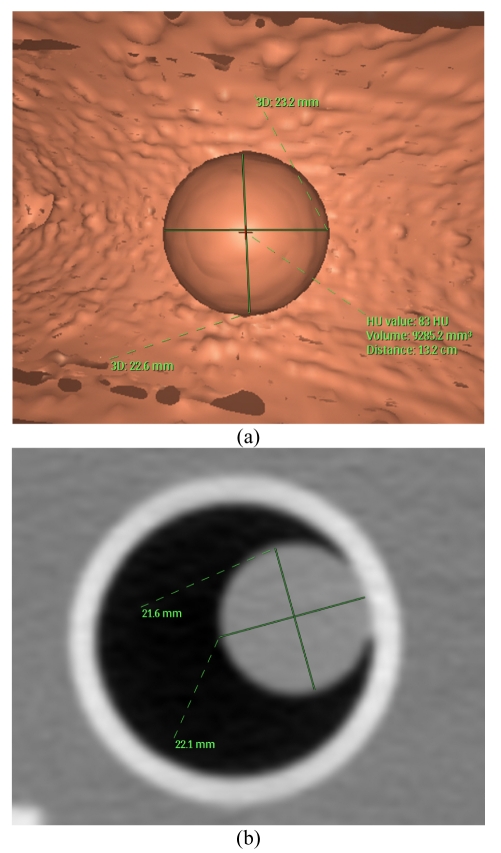
Sample measurements: (a) Image shows an axial 2D slice with the largest visible diameter (Diameter 1 = 22.1 mm) measured along with the orthogonal diameter (Diameter 2 = 21.6 mm); (b) Image shows a polyp on the 3D endoluminal view with two diameter measurements. The image also shows CAD volume measurements that are computed automatically.

Diameter 1 is the maximum diameter of a polyp measured by readers or CAD, and Diameter 2 is the measurement orthogonal to the maximum diameter. For 2D measurements, readers used axial 2D slices and the maximum diameter measurements were made on the slice with the largest visible diameter. The orthogonal measurement to the maximum diameter was recorded as Diameter 2. For 3D measurements, readers used the endoluminal view. Sample measurements are shown in Figure 5.

All polyps except one (3.2mm) were detected by Colon CAD in both thin and thick scans. There was no difference in the CAD standalone sensitivity between thick and thin slices, with CAD achieving 100% sensitivity for polyps ≥ 6 mm. The absolute mean error in automatic Colon CAD volume measurements for thin and thick slice scans were 8.7% and 6.8%, respectively. The difference in the CAD volume measurements between thick and thin slice datasets is not statistically significant (P>0.05). The absolute mean error for Colon CAD diametric measurements was lesser in thin slice datasets than thick slice datasets but the difference was not statistically significant. The absolute mean error for readers’ manual 2D and 3D measurements was in most cases lesser for thin-slice datasets compared to thick slice datasets. The absolute mean errors (%) for all measurements are shown in Table 3. The differences between measurement were found to be statistically insignificant (P>0.05). Almost all CAD and readers’ 3D measurements overestimated the size of polyps but it was noticed that the overestimation was relatively higher for small polyps. Both over- and underestimation of polyp sizes were observed in the readers’ 2D measurements.

**Table 3:** Results show the absolute mean errors for automatic measurements and manual measurements (2D and 3D) by readers when compared with actual polyp sizes.

Dataset Type	Orthogonal Diameters	Absolute Mean Error (%)				
		Colon CAD	Reader 1 (3D)	Reader 1 (2D)	Reader 2 (3D)	Reader 2 (2D)
Thin Slice	Diameter 1	7.85	9.96	5.74	12.21	5.93
	Diameter 2	4.99	8.46	4.23	11.55	10.54
Thick Slice	Diameter 1	9.59	12.03	6.15	16.49	5.43
	Diameter 2	5.94	15.04	3.62	14.05	10.05

Dataset Type	Absolute Mean Error (%) in Volume measurement by Colon CAD
Thin Slice	8.72
Thick Slice	6.78

The Bland-Altman analysis computed the interobserver agreement between the readers for repeat manual 2D and 3D measurements of simulated polyps. The mean difference between the observer measurements and the 95% Bland-Altman limits of agreement are shown in Table 4.

**Table 4:** Interobserver Agreement Analysis: Comparing manual 2D and 3D measurements from two readers on thick and thin slice datasets

Dataset Type	Orthogonal Diameters	3D		2D	
		Mean Difference	95% Bland-Altman Limits of Agreement	Mean Difference	95% Bland-Altman Limits of Agreement
Thin Slice	Diameter 1	0.11	1.8, -1.5	-0.87	0.8, -2.6
	Diameter 2	0.16	1.5, -1.1	-0.93	0.3, -2.2
Thick Slice	Diameter 1	0.43	1.3, -0.5	-0.96	0.1, -2.0
	Diameter 2	-0.15	1.1, -1.4	-0.6	1.1, -2.3

## DISCUSSION

The Colon CAD software for CT Colonography may potentially improve readers’ detection performance and reduce variability among readers [11]. The CAD software can be used in a concurrent reading (CAD findings highlighted during the radiologist’s primary read) or a sequential/second reading paradigm (CAD findings highlighted only after the radiologist’s primary read is complete). Some studies have suggested that CAD may benefit novice readers more than the experienced ones [12]. Colon CAD provided accurate size measurements for a wide-ranging size of phantom polyps, and results were comparable to the manual measurements made by two independent readers. The absolute % (mean) error for CAD and for the readers was relatively higher for smaller polyps (6-9 mm diameter) than large polyps (≥10 mm diameter). Overall, Reader 1, the expert in interpretation of CT colonography, had a smaller mean error than Reader 2. As mentioned above, polyps can be categorised based on their size (diameter) and three clinically relevant categories have been defined [1]. In our study, based on readers' and CAD measurements, no phantom polyp was misclassified in a larger or smaller size category due to over- or under-estimation of size respectively. But this may be due to the low number of sample polyps with sizes that are close to the polyp size categorical boundaries. This is an important issue since the recommended patient follow-up may change substantially as the size of the polyp detected increases [1]. For example, in some clinical settings, a patient with a single 6-9 mm polyp may be triaged to a follow-up CT colonography while a patient with a polyp above 10mm in diameter is generally considered to have an advanced adenoma for which optical colonoscopy would be recommended. Therefore, this finding needs further analysis with a larger sample size.

In our study, readers’ 2D measurements were more accurate than 3D measurements. All polyps except one (3.2mm) were detected by Colon CAD in both thin and thick scans. This polyp was missed in both thin and thick slice datasets. This may be due to the fact that the manufacturer default threshold for the Colon CAD algorithm is set at detecting polyps ≥6 mm. The polyp was visible to the naked eye in both the datasets since the reconstruction of both datasets were thin enough to reveal such small polyps. The size of the missed polyp being very close to this threshold could have been the reason for non-detection. In fact, after the study was completed, the 3.2mm polyp was shown to be detectable when the diameter threshold for CAD was reduced below the default used for the study. The clinical significance of detecting polyps in the 3-4 mm size range remains a subject of controversy. The published consensus proposal for reporting CT colonography considers colonic polyps less than or equal to 5 mm in diameter or so-called diminutive polyps as not clinically significant [1]. CAD achieved 100% sensitivity in detecting polyps ≥6mm in this phantom.

## LIMITATIONS

A typical CT Colonoscopy patient prep includes ingestion of a low-density barium suspension for tagging solid fecal residue and iodine-based contrast material for tagging the fluid that remains inside the patient’s colon. This helps in differentiating between polyps and other non-polyp material. The presence of tagged or untagged fluid/fecal material on or close to the polyps may affect the manual and automatic measurements. In this study design, this effect was not measured due to the limitations in the design of the phantom. Future studies to evaluate the effect of fluid and fecal material (tagged or untagged) in the colon on these measurements are needed. Due to the small sample size (number of phantom polyps) and use of only two dissimilar readers, we did not analyse inter- and intra-observer variability for the manual 2D and 3D measurements. Future studies with larger sample size are needed to perform this analysis. Lastly, the phantom used in this study is a simplistic approach to a real patient colon. To verify that these findings are reproducible in a clinical setting, a similar study is needed using a more complex phantom with a morphology that mimics colonic folds and flexures as well as non-spherical phantom polyps that mimic the irregular shapes of real polyps.

## CONCLUSION

CAD provided accurate size measurements (diameter and volume) for all simulated polyps. The CAD automated measurements were comparable to the two readers' manual measurements. This proves the ability of CAD to provide automated measurements of diameter which may help decrease interpretation time for CT Colonography. CAD was also very sensitive to the polyps that are considered clinically relevant. This study shows that CAD may prove to be beneficial for CT Colonography.
